# Using Video Ethnography and Stimulated Recall Interviews to Describe the Diagnostic Process in the Emergency Department

**DOI:** 10.1111/acem.70142

**Published:** 2025-09-03

**Authors:** Milisa Manojlovich, Caitlin Cassady, Sarah J. Parker, Ellie Davis, Charlotte Ahr, David Ryamukuru, Anna Wang, Kalyan Pasupathy, Hardeep Singh, Prashant Mahajan

**Affiliations:** ^1^ University of Michigan School of Nursing Ann Arbor Michigan USA; ^2^ Department of Emergency Medicine University of Michigan Ann Arbor Michigan USA; ^3^ University of Illinois at Chicago Chicago Illinois USA; ^4^ Center for Innovations in Quality, Effectiveness and Safety, Michael E. DeBakey Veterans Affairs Medical Center and Baylor College of Medicine Houston Texas USA

**Keywords:** diagnostic error, emergency service hospital, qualitative research, video‐audio media

## Abstract

**Objectives:**

Understanding how physicians make diagnoses is challenging because cognitive processes are unobservable and partly unconscious, making it difficult for physicians to describe how they arrived at a diagnosis. Physicians who work in emergency departments (EDs) are especially vulnerable to making diagnostic errors because the ED is a fast‐paced, dynamic setting where complex decision‐making occurs under severe time, information, and resource constraints. The purpose of our study was to describe how the diagnostic process evolves for ED clinicians in both pediatric and adult ED settings.

**Methods:**

We used a qualitative, video ethnography study design to capture in situ, real‐time ED physician practice for 11 participants from February 2022 to July 2023. Participants wore a head‐mounted video camera while providing care to ED patients, and in subsequent stimulated recall interviews, revealed their thinking throughout the diagnostic process.

**Results:**

We recorded 24.42 h of video overall (average 2.22 h per participant). We identified four major themes in the ED diagnostic process: (1) quality communication facilitates information flow, (2) cognition is complex and distributed across patients and the ED team, (3) artifacts can enhance the diagnostic process, and (4) there is a need to balance efficiency with safety and accuracy.

**Conclusions:**

Illustrating physicians' cognitive processes through video ethnography coupled with stimulated recall interviews helped advance our understanding of the diagnostic process and is a foundational step for identifying improvement opportunities.

## 1 Introduction

It is often difficult for emergency department (ED) clinicians to articulate how they arrived at a diagnosis [1]. Without the ability to describe how a diagnosis was made, the process remains opaque and susceptible to error. There are several aspects to the diagnostic process that make it difficult for clinicians to describe. Among them, three in particular stand out. First, diagnosis is an operationally complex cognitive process [2]. For example, in the ED, frequent interruptions disrupt cognition and may result in safety concerns, including prescribing errors [3] or breaks in tasks [4]. Second, cognition is tightly coupled to the context in which it occurs because it is distributed across people and settings, but this distinction often goes unacknowledged [5]. Third, diagnosis does not unfold in a linear process [6] and capturing nonlinear aspects of diagnosis is challenging.

For these reasons, current research methods, whether quantitative or qualitative, may be insufficient to comprehensively understand factors that affect the diagnostic process in the ED. A promising but underused methodology that very few research teams have adopted involves video ethnography coupled with stimulated recall interviews [1, 7]. Video‐recorded encounters capture events as they occur, reveal contextual influences on diagnosis, and can be reviewed multiple times [8]. Video‐based methods have the potential to identify patient, provider, and system‐related factors that affect diagnosis because making a diagnosis in the real world is influenced by all three [9, 10, 11]. Prospective filming of encounters as they actually occur, in combination with retrospective stimulated recall interviews, can help reveal clinicians' thinking at the time of the encounter [12, 13]. The video acts as a prompt to stimulate recall [14] and the interview gives clinicians an opportunity to discuss what they were thinking at the time. We extend the work done by Pelaccia and colleagues in this area by adapting their video method described as ‘own‐point‐of‐view’ where clinicians wear a small head‐mounted camera to capture their field of view [1] followed by stimulated recall interviews using video clips as prompts.

### 1.1 Theoretical Model

The theory of distributed cognition guided our work. Distributed cognition is a social cognitive theory that describes how cognition is dispersed across people, places, and things [15]. The theory is comprised of three main principles: the physical organization of work, information flow, and artifacts [16]. The physical organization of work refers to the structure of the environment in which the work takes place, which we called “workflow.” Information flows in a system through communication (a key aspect of the diagnostic process) [17], an interpersonal process that results in shared understanding between patients and clinicians as well as between clinicians from different specialties and roles. Artifacts are cognitive supports such as notes, tools, or electronic applications, including the electronic health record (EHR). We selected the theory of distributed cognition because of the focus on cognition in its many manifestations, the social nature of clinicians' work, and the context‐dependent nature of the work, particularly in the ED, which is uniquely dynamic and unpredictable compared to other settings [18, 19].

The purpose of our study was to describe how the diagnostic process evolves for ED clinicians in both pediatric and adult ED settings. Our approach consisted of asking four related questions: (1) What is the effect of patient/family input on the diagnostic process? (2) What are the effects of work environment factors (including culture, system) on the diagnostic process? (3) What are the effects of communication and interpersonal factors on the diagnostic process? and (4) What cognitive strategies do emergency medicine providers use to aid the diagnostic process?

## 2 Methods

We used a qualitative, video ethnography study design to capture in situ, real‐time ED physician practice from February 2022 to July 2023.

### 2.1 Setting and Participants

We video‐recorded interactions in two sites—an adult and a pediatric ED that were both part of the same academic Level 1 trauma center in the mid‐western United States. We included adult and pediatric EDs within the same health system to examine differences such as patient age, illness, interactions, sociocultural factors, or physical layout that may affect the diagnostic process. The comparison of adult and pediatric EDs in this study provides a unique approach for examining the influence of age‐related factors and intra‐institutional variation on diagnosis. After obtaining the approval of physician and nursing leadership in both pediatric and adult emergency departments, we used purposeful sampling to recruit attending level physicians who provided care to either adults or children in one of the two EDs across varying shifts (e.g., day, evening, night). Attending physicians were recruited via email from February 2022 to May 2023. Digitally signed informed consent was obtained prior to study participation.

We notified other emergency department staff of the study, anticipating that their images could be captured incidentally during interactions with the attending physician participants. The Institutional Review Board granted approval for this study (HUM00156261).

### 2.2 Video Procedures

Participants wore a head‐mounted micro camera that filmed their “own‐point‐of‐view” while they provided care to patients during a regularly scheduled ED shift. Two observers shadowed participants and took hand‐written field notes of their observations. We excluded interactions with patients requiring immediate intervention, patients who were prisoners, cases of suspected child abuse, or patients in rooms with special pathogen precautions (including COVID‐19). Participants independently identified other cases to exclude and notified research team members when the equipment needed to be removed or a recording stopped. We gave participants the opportunity to review their video either immediately after filming or later. We aimed to collect approximately 2 h of video‐recorded data from each participant, as this time duration provided opportunities to observe multiple interactions and in a past study did not place an undue burden on participants [20].

### 2.3 Post Video Editing

Members of the research team independently reviewed all video recordings to identify video clips where a participant seemed to be engaging in the diagnostic process, and we made note of the timestamps when that occurred. During weekly meetings, we discussed what each of us had seen and arrived at a consensus on which clips resulted in the most interesting examples of diagnostic thinking. We then combined similar clips into one of three theoretical model‐based questions that were discussed during interviews: (1) How does the provider arrange or organize their workflow, and what are the implications of that workflow pattern for diagnosis, (2) How does the EHR and other artifacts help or hinder the diagnostic process, and (3) How do interactions with others affect the diagnostic process?

### 2.4 Stimulated Recall Interviews

Following joint review of the video clips, the research team developed questions and associated probes to ask participants during individual follow‐up interviews to derive a deeper understanding of what was occurring. We chose the interview topics, because of our interest in answering research questions according to the theory of distributed cognition. Follow‐up interviews were conducted via Zoom, which was a convenient option for participants. Reserving a conference room on site for in person interviews was not feasible because of several logistical issues, including lack of space, parking restrictions, and lack of participant availability on certain days. We selected approximately 10 min worth of exemplar video clips for each interview to stimulate participant recall. Clips were introduced and then played for the participants, followed by the interview question and probes. Video clips ranged from 5 s to around 2 min long. The number of selected clips for each theoretical model‐based question ranged from 1 to 8. We asked probing questions after each clip. For example, to start Interview No. 1 we asked, “This first clip shows you picking up a new patient. How do you find out about new patients?” Occasionally, we played several short clips in a row before asking a question. All clips were collated in an Excel spreadsheet (Appendix S1). Each interview lasted approximately 1 h. The interviews were video‐recorded and professionally transcribed. The interview transcripts were then compared to the recording by a member of the research team to verify accuracy and completeness.

### 2.5 Coding and Rater Consensus

Transcripts were reviewed independently by research team members who met weekly to discuss initial impressions of each interview and generate codes for the codebook. Codes were generated deductively using the theoretical model and inductively as new codes were discovered throughout the coding process. For each of the first three interviews, four members of the research team independently coded the same transcript and then met as a group to discuss each code, achieve consensus, and develop consistency in the application of the codes. Pairs of researchers coded the remaining eight interviews, and the full team discussed updates to the codebook weekly. An audit trail was maintained to record changes.

### 2.6 Data Analysis

We used MAXQDA [21] to organize and analyze the data using interaction reports to develop a matrix and identify relationships among the categories that would address the research questions [22]. For each research question, the interaction reports were divided among the research team and individually reviewed and summarized before being discussed as a group. We did not use the field notes for analysis, although they were available.

## 3 Results

Eleven attending physicians participated in the study. We intended to recruit an equal number of physicians from each ED, but an 11th physician agreed to participate at the end of the recruitment period. Seven participants were male, and four were female. Years of experience in the ED ranged from 3 to 17 years. We completed five observations in the pediatric ED and six observations in the adult ED. We recorded 24.42 h of video overall (average 2.22 h per participant).

We identified four themes: (1) quality communication facilitates information flow, (2) cognition is complex and distributed across patients and the ED team, (3) artifacts can enhance the diagnostic process, and (4) there is a need to balance efficiency with safety and accuracy. The first three themes arose from deductive analysis while the fourth theme was derived inductively. However, not every theme was present in answering each research question. Each section below represents the answer to one of the four research questions.

### 3.1 What Is the Effect of Patient/Family Input on the Diagnostic Process?

Participants told us that patient and family input had a major effect on the diagnostic process, which appeared in each of the four themes. Even though the relationship between patients and providers was of short duration in the ED, many providers told us how important it was to the diagnostic process. Participants believed that patients were more willing to share information when there was trust and described strategies such as getting down to eye level with a patient and introducing levity when appropriate into a conversation to form a relationship (Table 1, row 1). Patients did not always share complete information about their symptoms, and without building rapport, providers perceived that they were less able to elicit information that could help make a diagnosis. Second, the theory of distributed cognition acknowledges the complexity of cognition and how it is distributed across patients and the ED team. For example, one provider said that reviewing test results with patients was “critical” to ED work because discussions of test results helped patients and families understand what was wrong and also what was not wrong (Table 1, row 2). Third, artifacts used by patients and family members influenced the diagnostic process. Video clips or pictures documenting symptoms provided by patients/family members helped the diagnostic process because the images were more objective and often more clearly demonstrated what the issue was than words alone (Table 1, row 3). Finally, patients and family members affected the balance of efficiency with safety and accuracy. Sometimes patients requested testing that would take a lot of time, and the provider had to decide the extent to which the additional testing would confirm a tentative diagnosis versus cause a decrease in efficiency by holding up patient flow through the ED (Table 1, row 4).

**TABLE 1:** Research Question 1: What is the effect of patient/family input on the diagnostic process?

Themes	Exemplar quotes
1. Quality communication facilitates information flow	This kind of [patient‐provider] interaction takes down all the barriers and all of the walls …this just makes it, “Hey, you know, I'm just a guy that cares about you, that wants to see you do okay, so tell me what's going on” … so I think it builds that rapport a whole lot quicker than if I were just to go in there with a whole bunch of bullet point questions, answer my questions, and then I'm out of here. (Interview 5)
2. Cognition is complex and distributed across patients and the ED team	I think that patients want to know, and to me, that's exactly why they're there. Most of the time they're not there just to know what they have. They also wanna know what they don't have, because sometimes we can't really tell them what they have. So to me, this is just the critical part of what we do, and it's closing the loop in the diagnostic cycle. (Interview 11)
3. Artifacts can enhance the diagnostic process	It [photo/video shared by family] can be super helpful … even if it's just like, a still picture of things …parents will have it for here's how my child was working hard to breathe earlier today … sometimes they'll even have video of the fall that the kid had … and it's really helpful to just kind of take the subjectivity out of what the parents are telling us. (Interview 3)
4. Balancing efficiency with safety and accuracy	Some of what we do is meeting the expectations of patients… especially when I think that there is diagnostic equipoise, I am okay with doing what patients want, even if it's not what I would've done for myself…But when it puts patients at risk, or increases the risk to patients, or puts other patients and then the emergency department capacity at risk unfairly, then I draw the line. (Interview 11)

### 3.2 What Are the Effects of Work Environment Factors (Including Culture, System) on the Diagnostic Process?

Work environment factors had an effect on the diagnostic process and included cultural and system factors as well as characteristics of the physical ED space. Work environment factors were apparent in three themes. Since our study was conducted in an academic medical center, our participants were responsible for training residents, medical students and new physician assistants. The unique cultural characteristics of medical training in the clinical arena influenced how and when communication occurred (Table 2, row 1). Clinician resources, such as decision rules, UpToDate, or PubMed, were examples of artifacts that could be helpful in the diagnostic process. These resources reminded participants of rare conditions or diagnoses and helped them decide treatment plans, interpret lab results, or determine whether testing was necessary (Table 2, row 2). However, the need for efficiency was the core driver of the diagnostic process in the ED and could override the number and type of diagnostic tests that were ordered. For example, during busy times, participants knew that low acuity patients typically had longer to wait to be seen so they tended to place many orders at the same time in what was called a “shotgun” approach. The result of this approach was to reduce wait times for test results which improved efficiency. However, there were certain tests that could limit efficiency in the ED, such as magnetic resonance imaging (MRI). Participants were aware of the potential for life‐threatening diagnoses and would order appropriate tests to rule them out, because of the ED doctrine of “worst is first” (Table 2, row 3). With high patient volume, the configuration of the physical space was altered such that stretchers were placed in the halls. These “hall beds” were meant for low acuity patients but affected efficiency. For example, some patients became sicker while in a hall bed and needed to be moved to a private cubicle where there were more supplies, but moving patients took time and staff effort that limited efficiency.

**TABLE 2:** Research Question 2: What are the effects of work environment factors (including culture, system) on the diagnostic process?

Theme	Exemplar quotes
1. Quality communication facilitates information flow	PAs and residents will be seeing and … wanting to present patients to you, right? But if you're busy … in a room and it's taking a lot longer in that room, they cannot present that patient to you because you're not available. And so that stops workflow … many of them still …have questions or want a second opinion or just aren't comfortable, or it may be a challenging diagnosis. So … they still want to staff those with me, so … I have to be available. (Interview 1)
2. Artifacts can enhance the diagnostic process	I would say it definitely helps with the diagnostic process … when we're using UpToDate, it's … a great resource. It's very reliable … but it's not super, like deep dive into anything … if there's debate about appropriate treatment plans … I'll go more to like the PubMeds … to see if I can find a meta‐analysis, to see if there's a consensus that's up to date in the literature. (Interview 3)
3. Balancing efficiency with safety and accuracy	Most of those [evidence‐based] rules go out the window when you're really busy because you're just going to get an X‐ray … and not think about the clinical decision making rule … Our doctrine is worst is first in the ED. And so if … someone says the worst symptoms that are consistent with some terrible diagnosis, we're probably going to test for it… Whereas … if it is not like a life or death … diagnosis … they probably have some more wiggle room in terms of … their diagnostic testing. (Interview 6)

Abbreviations: ED, emergency department; PA, physician assistant.

### 3.3 What Are the Effects of Communication and Interpersonal Factors on the Diagnostic Process?

Communication and interpersonal factors appeared in three themes and played a crucial role in the diagnostic process (Table 3). Communication mediums varied depending on the urgency and complexity of information. For example, paper communication was sometimes faster in communicating test results than electronically released results, especially for point‐of‐care tests such as arterial blood gas results. Gathering information through multiple communication mediums reportedly helped the participant reduce communication errors (Table 3, row 1). Photos could aid information gathering and were perceived as being more efficient than verbal communication for certain exam findings (Table 3, row 2). Participants identified several communication barriers that they believed affected the diagnostic process by disrupting the balance between efficiency and safety/accuracy. These barriers included interruptions from other clinicians, lack of physical proximity to the patient and other members of the care team (when patients were in areas where the provider was not stationed), language differences between patients and providers, and lack of trust in other clinicians. Participants developed strategies to address such barriers (which represent interpersonal factors) by taking time to learn about new staff so that they could deliver safe and high‐quality care as a team. Although it took time to develop trust, participants believed that the time invested in building relationships with other members of the team paid off in terms of efficiency gains later on (Table 3, row 3). We found that participants prioritized staffing patients with physician assistants and residents because they believed that otherwise overall workflow slowed.

**TABLE 3:** Research Question 3: What are the effects of communication and interpersonal factors on the diagnostic process?

Theme	Exemplar quotes
1. Quality communication facilitates information flow	Our consults will often use it [chat function] to get in touch with me as well. And if that's how they do it, I will use the chat function to respond to them. Um, there again, I would prefer phone calls just so it's a more organic back and forth. Um, but…like, especially our psych consults, um, will…they seem to be most, uh, heavy users in the chat function. Um, and it works. I mean, you know, it's, it is nice in the sense that there's less communication errors. (Interview 3)
2. Artifacts can enhance the diagnostic process	You can describe the rash all you want, but when you see it in picture, it's completely different. So I tend to advocate for… a snapshot of what it looked like at discharge. So, um, I think it helps providers in the future. I think it also documents what you're seeing … or that's why you thought the differential was such is because, you know, photos are pretty powerful. (Interview 1)
3. Balancing efficiency with safety and accuracy	Many of them [new PAs] don't have, um, really quite similar experiences to be able to optimally function in our emergency departments … and that really slows me down. …I don't want there to be a bunch of PA turnover. I think that is negative to our ability to optimally care for patients. And so, I want to try to do a little investment in that relationship building … Is there something this person needs to function optimally today so that we can continue to do good patient care? (Interview 2)

Abbreviation: PA, physician assistant.

### 3.4 What Cognitive Strategies Do Emergency Medicine Providers Use to Aid the Diagnostic Process?

Unsurprisingly, cognitive strategies were apparent in all four themes, suggesting a large effect on the diagnostic process (Table 4). Cognitive strategies were described as bidirectional between clinicians and patients and their families, and participants used strategies to make their thinking explicit, such as discussing differential diagnoses with other clinicians (Table 4, row 1). A strategy some participants mentioned was discussing or writing out a full differential diagnosis because, in doing so, the possibilities became explicit and could be shared. Providers told us how explicitly documenting the differential diagnosis on a piece of paper or in the EHR was important and also helpful for future providers to know what the initial concerns were. This strategy helped reduce the chance of “missing things” defined as a piece of information from the patient or diagnostic testing that does not get processed or discussed (Table 4, row 2). Artifacts in this category included cognitive aids such as paper lists and notes in the EHR (Table 4, row 3). Providers acknowledged that they must balance efficiency with accuracy and the likelihood of missing things. When there was diagnostic uncertainty, it was important for the clinical team to wait and gather more information if possible. However, sometimes waiting was not an option because of overcrowding and the need to prepare resources for the next patient (Table 4, row 4). Although we did not compare responses from participants in the adult ED to those of the pediatric ED, themes were found across both settings as reported above, suggesting that any difference between the sites was minimal.

**TABLE 4:** Research Question 4: What cognitive strategies do emergency medicine providers employ to aid the diagnostic process?

Theme	Exemplar quotes
1. Quality communication facilitates information flow	I could order that test, he [physician assistant] could order that test, but like making sure like that process happens … and he's kept in the loop as well … as like data comes back to him too, like he understands my own thinking and we're sort of contributing, you know, to the diagnostic process equally … so we're all … maximizing the data that we're getting back collectively as a team too. (Interview 10)
2. Cognition is complex and distributed across patients and the ED team	Sometimes when we're going quickly, I find people don't actually explicitly talk about the differential, and then we end up missing things… Or … it doesn't actually rise to the conscious to actually have us consider it in the patient's workup. (Interview 2)
3. Artifacts can enhance the diagnostic process	What I will also do is I'll write down their name, age and at least chief complaint on a piece of paper to go with me just as … a little cognitive aid, just a reminder. Then, on that piece of paper, too, I'll just make a little checkbox if there's any specific to do. (Interview 3)
4. Balancing efficiency with safety and accuracy	You could be the greatest clinician and not make a mistake, but if you're only seeing 30% of patients, at what cost, right? And so … that's what makes, I think, emergency medicine that challenging. Who's the sick one? Who's gonna get worse? (Interview 1)

## 4 Discussion

The purpose of our study was to describe the diagnostic process in both pediatric and adult ED settings to better understand how the diagnostic process evolves for ED clinicians. We used the theory of distributed cognition to guide our qualitative approach and found that the diagnostic process evolves through a complex combination of four overlapping themes: (1) quality communication facilitates information flow, (2) cognition is complex and distributed across patients and the ED team, (3) artifacts can enhance the diagnostic process, and (4) there is a need to balance efficiency with safety and accuracy.

Our study makes several unique contributions to the literature. First, the majority of studies related to diagnosis have relied on retrospective approaches, which are influenced by hindsight bias [23]. We minimized this bias by taking a prospective approach, as did Pelaccia [1, 10], and using video ethnography in an adaptation known as “own‐point‐of‐view” video to collect data from 11 ED physicians during actual patient care. Second, we did not interpret the video produced by participants, which would have called into question the ecological validity of our findings [17]. Rather, we shared video clips during video‐stimulated recall interviews that allowed participants to describe cognitive processes in their own words. Third, we did not notice differences in the diagnostic process for participants in the adult versus pediatric emergency departments, and to our knowledge, this may be the only study where data were collected concurrently from both types of EDs. Finally, by developing a matrix to identify interactions among themes during analysis, our findings may more closely represent the complexity of the diagnostic process in the ED [22].

Participants facilitated quality communication by building rapport with patients and their families, teaching resident physicians and new staff, selecting appropriate communication mediums, and discussing their thoughts with team members. The importance of building rapport, or trust, is consistent with other research on the relationship of trust between patients and caregivers [24, 25, 26]. Although establishing rapport with patients, families, and other clinicians initially took time, we heard how the investment was perceived to be important.

Our finding that artifacts impact the diagnostic process has also been reported by others. Patient‐generated health data, such as photos or videos that patients and families bring to the ED encounter, can support clinicians in diagnosis and empower patients in sharing their personal experiences [27]. Although the EHR is readily accessible, participants described personal paper notes as being more personalizable and portable, which is consistent with other studies [28]. What our study adds is that artifacts such as photos were reported to promote efficiency by combining symptoms and visual data into a single image that took the subjectivity out of what participants were being told.

The acknowledgment that the ED “can only go so far” as reflected by several participants illustrates the importance of Pulia et al.'s call to acknowledge and communicate diagnostic uncertainty which is inherent to the ED diagnostic process during transitions of care [29]. Diagnostic uncertainty could be exacerbated by interruptions which are known to affect cognition [3, 30, 31, 32]. Participants reported how their cognition could be disrupted by interruptions which then slowed them down and could adversely affect efficiency and the diagnostic process more generally.

The need to balance efficiency with safety and accuracy has been noted by others. Dean et al. also discussed the tension between efficiency versus rapport and comprehension, in the context of physician–patient communication [33]. What this study adds is how the diagnostic process changes when the ED is busy. Participants described how the need for efficiency and throughput superseded evidence‐based clinical guidelines during times of overcrowding.

Few studies have explored how diagnosis is achieved in the ED using video research methods. To the best of our knowledge, there has been very limited use of head‐mounted cameras and postfilming stimulated recall interviews to understand ED physician thinking during the diagnostic process. We adapted Pelaccia's method of having physicians wear head‐mounted cameras to film their “own‐point‐of‐view,” [1] but we did not focus on the specific topic of generating and evaluating diagnostic hypotheses as did Pelaccia. Rather, we used “own‐point‐of‐view” to gain a better understanding of ED physicians' reasoning during the diagnostic process. Our analysis builds on work done by Pelaccia [1] by offering additional insights into physicians' cognitive processes beyond initial hypothesis generation in the ED.

There are several limitations to this study. First, our study setting was two EDs (adult and pediatric) within one large, academic medical system. The perspectives of ED physicians, therefore, may vary based on differences in organizational characteristics, such as limited diagnostic resources in a smaller or nonacademic institution. Second, the average length of time between video recording and interview was 59 days (range 39–87 days) because of the amount of time needed to edit videos and have the research team meet to come to consensus on clips to include during interviews. Also, scheduling interviews was driven by limited availability of busy participants. As a result, participants' ability to recall their thought process related to specific scenarios was potentially limited. However, physician comments such as “I don't remember this case” or “I remember this one” made during interviews and captured in transcripts were not associated with the time delay between when the video was recorded and the interview. Although we found that participants were able to share important insights into their cognitive processes around diagnosis in the ED, we used an indirect method of obtaining evidence of cognitive activity and cannot be certain that their words represented thoughts they had at the time of filming. The camera, even one as small and unobtrusive as the one we used, becomes part of the research and introduces the possibility of a Hawthorne effect, although such effects do appear to be small [34, 35, 36]. Finally, although we video‐recorded conversations with patients, they were not the focus in the study. Future studies could consider the patient perspective in the diagnostic process. Despite these limitations, this study advances what is known about the ED diagnostic process through our use of video‐stimulated recall, which promoted a deeper exploration of the cognitive process beyond what is possible with other observational techniques.

Two implications of our findings for emergency medicine practice stand out. First, the physical environment could be configured so that physicians, nurses, and others who work in the ED share space, which would facilitate direct conversations. Human decisions are often opportunistic and influenced by the “many distributed interactions” that occur between people [37], so sharing space could make such serendipitous interactions more likely. Second, systematic cross‐checking of information between two ED physicians could be introduced as a way to reduce adverse events and near misses, as was reported in a study done in France [38]. Cross‐checking involves one physician presenting a case to another and receiving feedback on it, with the premise that the fresh perspective brought by the second physician could yield an insight that the first physician had not considered.

## 5 Conclusion

The use of video ethnography coupled with simulated recall interviews helped us understand several aspects of the diagnostic process and how they interact with each other. The theory of distributed cognition was well suited to our research question, and video ethnography coupled with stimulated recall interviews revealed aspects of diagnosis that otherwise would have remained hidden. Developing a better understanding of how physicians make diagnoses in the ED is the first step toward identifying opportunities for improving the diagnostic process.

## Author Contributions

P.M., K.P., and H.S. conceived the study, secured funding, and reviewed drafts for critical content. M.M. developed the methodology, supervised data collection and analysis and wrote the original draft. A.W. participated in data collection. C.C., E.D., C.A., D.R., and S.J.P. participated in data collection and analysis. All authors contributed to the review and editing of drafts. S.J.P. was responsible for the management and coordination of the study.

## Disclosure

The authors are solely responsible for this document's contents, findings, and conclusions, which do not necessarily represent the views of AHRQ. Readers should not interpret any statement in this report as an official position of AHRQ or of HHS. None of the authors has any affiliation or financial involvement that conflicts with the material presented in this report.

## Conflicts of Interest

The authors declare no conflicts of interest.

## Supporting information


**Data S1:** acem70142‐sup‐0001‐DataS1.xlsx.

## Data Availability

Certain data that support the findings of this study are available on request from the corresponding author. The data are not publicly available due to privacy or ethical restrictions.
